# Effect of BMI on the value of serum progesterone to predict clinical pregnancy outcome in IVF/ICSI cycles: a retrospective cohort study

**DOI:** 10.3389/fendo.2023.1162302

**Published:** 2023-04-19

**Authors:** Zhaoyang Shen, Xiaoyan Luo, Jianming Xu, Yuqing Jiang, Wenhui Chen, Qingling Yang, Yingpu Sun

**Affiliations:** ^1^Center for Reproductive Medicine, The First Affiliated Hospital of Zhengzhou University, Zhengzhou, China; ^2^Henan Key Laboratory of Reproduction and Genetics, The First Affiliated Hospital of Zhengzhou University, Zhengzhou, China; ^3^Henan Provincial Obstetrical and Gynecological Diseases (Reproductive Medicine) Clinical Research Center, The First Affiliated Hospital of Zhengzhou University, Zhengzhou, China

**Keywords:** IVF/ICSI, progesterone, hCG, obesity, clinical pregnancy

## Abstract

**Background:**

Numerous research have investigated the predictor role of progesterone (P) level on the human Chorionic Gonadotropin (hCG) trigger day of assisted reproductive technology (ART) outcomes. However, the relationship of progesterone levels on hCG day to clinical pregnancy outcomes in IVF/ICSI cycles for patients with different BMI groups is still elusive. This study aimed to investigate the effects of progesterone elevation on triggering day on clinical pregnancy rate (CPR) of IVF/ICSI cycles in patients with different female BMI.

**Methods:**

We conducted a retrospective cohort study included 6982 normal-weight parents (18.5Kg/m2≤BMI<25Kg/m2) and 2628 overweight/obese patients (BMI≥25Kg/m2) who underwent fresh day 3 cleavage embryo transfer (ET) in IVF/ICSI cycles utilizing GnRH agonist to control ovarian stimulation.

**Results:**

The interaction between BMI and P level on triggering day on CPRs was significant (*p*<0.001). The average level of serum P was reduced with the increase in maternal BMI. Serum P adversely affected CPR in distinct BMI groups. In the normal weight group, CPRs were decreasedas serum P concentrations gradually increased (*p*<0.001 for overall trend). The CPRs (lower than 65.8%) of progesterone level > 1.00 ng/ml on triggering day were significantly lower than that (72.4%) of progesterone level <0.5 ng/ml. In the overweight/obese group, CPRs showed a decrease statistically with progesterone levels of ≥2.00 ng/ml compared to progesterone levels of <0.5 ng/ml (51.0% VS. 64.9%, *p*=0.016). After adjusting for confounders, progesterone elevation (PE) negatively correlated with CPRs only in the normal weight group (OR: 0.755 [0.677–0.841], *p*<0.001), not in the overweight/obese group (*p*=0.063).

**Conclusion:**

Women with higher BMI exhibited a lower progesterone level on triggering day. Additionally, PE on hCG day is related to decreased CPRs in GnRH agonist IVF/ICSI cycles with cleavage embryo transfers regardless of women’s BMI level (normal weight VS. overweight/obesity).

## Introduction

Progesterone elevation (PE) on triggering day has become an important and controversial predictor of clinical pregnancy outcomes during IVF cycles since its first investigation in 1990 (1). As stressed by a great many researchers, premature luteinization and serum PE on trigging day have a detrimental influence on clinical pregnancy outcomes of IVF/ICSI cycles (2, 3). A meta-analysis for 63758 IVF cycles showed PE on triggering day caused reduced likelihood of positive pregnancy by almost 10% in fresh cycles (4). Meanwhile, another study demonstrated that the adverse influence of PE on clinical pregnancy outcome was only observed in poor to normal ovarian responders (5). However, some publications reported the opposite conclusions (1, 6). A consensus has been established that early PE advances the endometrial receptivity window causing endometrium-embryo asynchrony and adverse pregnancy outcomes (7). Studies of gene expression and epigenetic modification of endometrium on hCG day subsequently confirmed these results (8, 9). Many factors affect progesterone levels on triggering day, including the number of follicles and dosage of exogenous gonadotropins(Gn) (10). As compared to other abnormal reproductive states, obese women exhibit unique patterns of hypothalamic-pituitary-ovarian axis function. Furthermore, obesity has been demonstrated to be closely linked to reduced levels of progesterone, which can lead to menstrual irregularities and infertility (11–13). However, little was known about the serum P values on hCG day playing as predictor role of clinical pregnancy outcomes in overweight and obese patients in IVF/ICSI cycles.

As the prevalence of excess weight and obesity has increased to affect reproductive-age couples, overweight and obese women may encounter endocrine and metabolic problems, including menstrual dysfunction and anovulation resulting from the altered pulsatile secretion of gonadotrophin-releasing hormone(GnRH) and sex hormone binding globulin (14, 15). Considering these alterations, the endocrinological profiles of androgens, luteinizing hormone (LH), and other hormones like insulin and leptin in overweight and obese patients differ from that in normal population (14, 16). Overweight/obese women have lower levels of LH and FSH, P metabolites, and estradiol metabolites in their daily urine samples than the normal-weight population (12). Meanwhile, an increasing number of researches have indicated that pharmacokinetics of FSH and hCG preparations being affected by bodyweight, and increased BMI significantly and negatively influences clinical outcomes in ART cycles, including more gonadotrophins required, fewer oocytes obtained, reduced good-embryo rate, higher cycle cancellation rate, and miscarriage rate, lower clinical pregnancy rate and live-birth rate (17–20).

Progesterone at the appropriate level facilitates the endometrial transition from the proliferative phase to the secretory phase, which is necessary for embryo implantation and growth. Increased P may be a significant negative factor for pregnancy outcomes. At present, in published reports, the debate over the influence of PE on hCG day on pregnancy outcomes has primarily focus on different ovarian stimulation protocols (GnRH antagonist or agonist protocols) (21), stages of embryos transferred (cleavage stage embryo or blastocyst) (22) and different ovarian responses (low, intermediate and high responses) (23). The relationship of P level on hCG day to clinical pregnancy outcomes in IVF/ICSI cycles for patients based on BMI is still unknown.

The study aimed to figure out the relationship of P level on triggering day to CPR of GnRH agonist IVF/ICSI cycles based on distinct BMI populations. And we also analyze the thresholds where PE contributes to low CPR categorized in patients with different BMI.

## Materials and methods

### Patients and research design

A retrospective, single-center cohort study was conducted in Reproductive Medicine Center, First Affiliated Hospital of Zhengzhou University. The study has been reviewed and approved by the Ethics Committee of the First Affiliated Hospital of Zhengzhou University. Ethical approval number: 2022-KY-1099. Data were retrieved from 8996 fresh autologous IVF/ICSI-ET cycles from January 2017 to July 2021. We only recruited women who measured up to the following criteria. Inclusion criteria include: (1) 20-40 years old; (2) Only the first IVF/ICSI cycle followed by day 3 embryo transferred was included; (3) COS protocol was ultralong GnRH agonist. Exclusion criteria include: (1) The basal FSH concentration ≥10 mIU/ml; (2) Donor cycles; (3) Patients with uterine malformation including congenital uterine dysplasia, adenomyosis, endometriosis, uterine fibroids, endometrial polyps, and intrauterine adhesions; (4) Patients with recurrent spontaneous abortion; (5) Preimplantation genetic testing was performed. Patients’ clinical characteristics and cycle parameters were input into the hospital database. Based on BMI criteria (2000) of the World Health Organization, the patients were coarsely split into the normal weight group (18.5Kg/m^2^≤BMI<25Kg/m^2^) and the overweight/obese group (BMI≥25Kg/m^2^).

### Ovarian stimulation

Pituitary down-regulation was carried out by a follicular phase long-acting GnRH-agonist long protocol. Before ovarian stimulation, all patients received a long-acting GnRH-a (Triptorelin acetate) at a dose of 3.75mg intramuscularly on the day 2-3 of menstruation. After 28 days, ovarian stimulation was initiated if FSH, LH and E2 levels, follicle diameter, and number met the downregulation criteria according to ultrasound and sex hormone examination. The initial dosage of gonadotropin (recombinant FSH, Merck Serono) was 75-300 IU/d as per basal hormone levels, BMI, antral follicle counts and age. The dosage could be adjusted after monitoring follicle growth and endometrial thickness with transvaginal ultrasound and determining serum E2 levels. When the most prominent two follicles reached 17 mm, 10000 IU recombinant-hCG (Serono, Switzerland) was administered intramuscularly for triggering oocyte maturation. We retrieved oocytes approximately 35-37h following triggering by transvaginal ultrasound aspiration, followed by IVF/ICSI as previously described (24, 25).

### Pregnancy diagnosis

Embryos were cultured for 3 days in a carbon dioxide incubator at 37°C with 6% CO2, and their quality was graded using Peter cleavage embryo scoring system (26). Only embryos of grades I and II were viewed as high quality. Then, we transferred one or two high-quality embryos to the patient’s uterus. On the 14th day after embryo transfer, serum β-hCG levels were determined. Serum β-hCG levels>50IU were considered positive and diagnosed as biochemical pregnancy. Thirty-five days after embryo transplantation, transvaginal ultrasound was used to confirm clinical pregnancy by observing positive fetal cardiac activity and the gestational sac.

### Outcome evaluation

The clinical pregnancy is referred to as the presence of a gestational sac confirmed by ultrasound 35 to 45 days after embryo transfer. CPR=the number of clinical pregnancy cycles/the number of all embryo transfer cycles.

### P assessment immunoassay

Serum progesterone was detected between 8 and 12 a.m on days 2-3 of menstrual cycle, during ovarian stimulation together with on triggering day. Electrochemical luminescence immunoassay was employed for progesterone level measuring with detection sensitivity and assay variation of 0.15ng/mL and<4%, respectively.

### Statistical analysis

SPSS 25.0 (SPSS Inc, IBM Corp, USA) was adopted to conduct statistical analysis. Mean ± SD was presented for continuous variables, and an independent t-test or Kruskal-Wallis test was utilized for comparing fundamental and clinical characteristics among groups. Proportions or percentages were presented for categorical variables, and Chi-square test was utilized in comparing laboratory and clinical outcomes among groups. The interaction between BMI and P level on hCG day on CPRs was examined among all parents. To avert bias in results resulting from assumption that the relation of serum P levels to CPRs might not be linear, we divided all parents into eight different groups as per serum P level on triggering day: <0.50, 0.50-074, 0.75-0.99, 1.00-1.24, 1.25-1.49, 1.50-1.74, 1.75-1.99 and ≥2.00 ng/ml (22, 27, 28). We computed CPRs for every P interval and implemented Mantel-Haenszel test for trend analysis. Moreover, we calculated OR and 95% confidence interval (CI) of CPRs for every P interval to identify the P threshold via comparing each group with P <0.50 ng/ml group, then made pairwise comparisons among adjacent groups. We further built a multivariate logistic regression model to check if progesterone has preferential impacts on clinical pregnancy rates. In a multivariable regression model, we included variables as below: Age, BMI, infertility duration, AFC, basal FSH, basal E2, AMH, Gn dosage, days of stimulation, E2 on trigger day, and P on trigger day. *p*<0.05 denotes statistically significant.

## Results

8996 IVF/ICSI cycles in total were analyzed. To estimate whether there is an influence of BMI on the effect of P on clinical pregnancy outcomes, an interaction analysis was conducted to examine the relationship between BMI and serum P level on clinical pregnancy outcomes in all cycles. Our results indicated BMI possessed a significant moderating influence on the relation of P level on triggering day with clinical pregnancy (**Table 1**).

**Table 1:** The interaction analysis between BMI and progesterone level on the day of hCG on CPRs.

Factors	OR	95% CI	*p* value
BMI	0.991	0.977-1.005	0.218
Progesterone	0.730	0.677-0.788	<0.001
BMI·Progesterone	0.987	0.983-0.990	<0.001

BMI, body mass index.

**Figure 1:**
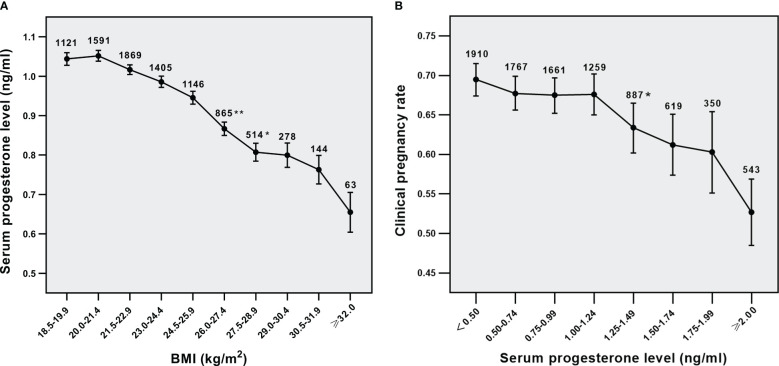
Relationship analysis. **(A)** Relationship between BMI and serum P levels in all parents, **(B)** Relationship between serum p levels and CPRs in all parents. ^*^p<0.05 and ^**^p<0.01 for comparison with previous P group; Data are expressed as mean ± SE or the OR (95% CI); SE, standard error; OR, odds ratio; CI, confidence interval.

As shown in **Figure 1A**, the average level of serum P was reduced with the increase in maternal BMI. Serum P level was negatively related to clinical pregnancy rate in all women (**Figure 1B**, *p*<0.001 for overall trend). Moreover, a statistically significant decrease in CPRs was seen while the serum P level was equal to 1.25 ng/ml at least, suggesting that serum P concentration of 1.25ng/ml was a possible cutoff level above which P was adversely affecting clinical pregnancy. These results indicate that BMI has an impact on the relationship between serum P level and clinical pregnancy outcomes.

The main baseline characteristics and pregnancy outcomes are presented in **Table 2**, according to the two BMI groups. Basal P and AMH levels between the two groups (*p*>0.05) showed no obvious distinction, while basal FSH and E2 levels of the overweight/obese group were lower than those of the normal weight group (*p <*0.001). The P level on hCG day was significantly higher in the normal weight group (1.0 ± 0.6) than in the overweight/obese group (0.9 ± 0.5) (*p*<0.001). Total Gn dosage and days of stimulation of the overweight/obese group were higher, and 2PN oocyte rate and high-quality embryo rate were inferior to the normal weight group (all *p <*0.05). In contrast, clinical pregnancy rate, implantation rate, miscarriage rate, or live birth rate between groups showed no obvious distinction (all *p*>0.05).

**Table 2:** Baseline characteristics and pregnancy outcomes according to female BMI.

	18.5≤BMI<25	BMI≥25	*p* value
Variables	N=6368	N=2628	
Female age (y)	30.7 ± 4.2	31.0 ± 4.4	0.020
Female BMI (kg/m²)	21.8 ± 1.7	26.8 ± 1.3	<0.001
Infertility duration (y)	3.7 ± 2.7	4.3 ± 3.1	<0.001
AFC	13.9 ± 6.1	15.1 ± 6.7	<0.001
Basal FSH (mIU/ml)	6.6 ± 1.5	6.3 ± 1.5	<0.001
Basal E2(pg/ml)	42.5 ± 25.3	38.6 ± 27.8	<0.001
Basal P (ng/ml)	0.5 ± 1.2	0.5 ± 1.0	0.312
AMH (ng/ml)	3.3 ± 2.5	3.4 ± 2.6	0.138
Gn dosage (IU/L)	2514.0 ± 964.4	2940.2 ± 981.8	<0.001
Days of stimulation	13.2 ± 1.9	13.7 ± 2.4	<0.001
E2 on hCG day (pg/ml)	2950.3 ± 1527.9	2434.1 ± 1334.9	<0.001
P on hCG day (ng/ml)	1.0 ± 0.6	0.9 ± 0.5	<0.001
Endometrial thickness on ET days (cm)	12.4 ± 2.7	12.5 ± 2.8	0.228
Oocytes retrieved	11.6 ± 4.7	11.7 ± 4.9	0.672
2PN oocyte rate (%)	61.2% (44022/71959)	59.8% (17896/29921)	<0.001
Cleavage rate of 2PN oocytes (%)	98.6% (43395/44022)	98.6% (17642/17896)	0.962
High-quality embryo rate (%)	63.6% (28020/44022)	62.2% (11126/17896)	0.001
Number of embryos transferred (n)	1.89 ± 0.31	1.86 ± 0.34	0.001
Implantation rate (%)	47.8% (5755/12030)	47.8% (2307/4894)	0.810
Clinical pregnancy rate (%)	66.3% (4222/6368)	65.1% (1612/2628)	0.293
Miscarriage rate (%)	7.4% (474/6368)	8.6% (226/2628)	0.063
Live birth rate (%)	57.5% (3661/6368)	55.4% (1455/2628)	0.064

Datas are presented as mean ± standard deviation (x ± SD). BMI, body mass index; AFC, antral follicle count; FSH, follicle-stimulating hormone; E2, estradiol; P, progestogen; AMH, anti-müllerian hormone; Gn, gonadotropin.

**Figure 2:**
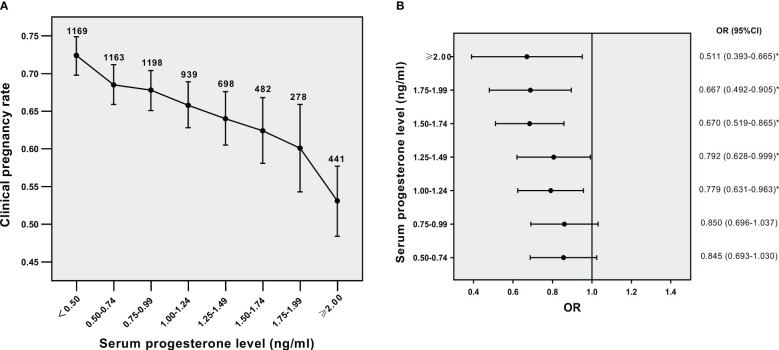
Relationship between serum P levels and CPRs in the normal weight group **(A)** Relationship between serum P levels and CPRs, **(B)** CPRs according to serum P levels. ^*^p< 0.05 for comparison with the lowest P group; Data are expressed as the OR (95% CI) for each serum P levels compared with the lowest P group (<0.5 ng/ml); OR, odds ratio; CI, confidence interval.

**Figure 3:**
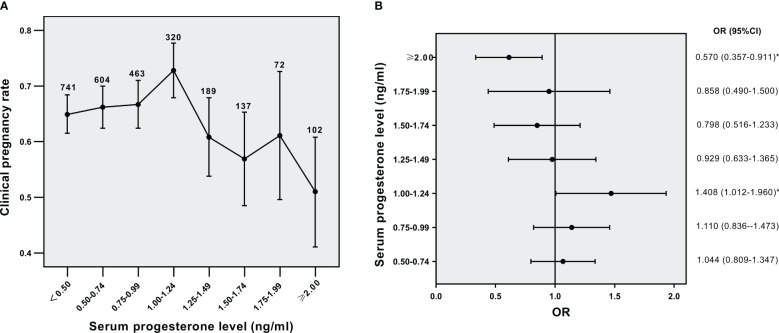
Relationship between serum P levels and CPRs in the overweight/obese group **(A)** Relationship between serum P levels and CPRs, **(B)** CPRs according to serum P levels. ^*^p< 0.05 for comparison with the lowest P group; Data are expressed as the OR (95% CI) for each serum P levels compared with the lowest P group (<0.5 ng/ml); OR, odds ratio; CI, confidence interval.

Then we analyzed the effect of serum PE on triggering day on CPRs in different BMI subgroups. The relation of serum P level to CPRs in the normal weight group was shown in **Figure 2A** (*p*<0.001 for overall trend). As the concentrations of serum P gradually increased, CPRs were decreased. The OR for CPRs in each serum P group compared to that of the serum P <0.5 ng/ml group was shown in **Figure 2B**. The five groups with P levels ≥1.00 ng/ml showed significantly lower CPRs than their lowest counterparts. The concentration of progesterone at 1.00 ng/ml might denote a threshold level exceeding which P has a significantly detrimental impact on CPRs in the normal-weight population. The relation of serum P level to CPRs in the overweight/obese group was shown in **Figure 3A** (*p*=0.016 for overall trend). The OR for CPRs in each serum P group compared to the serum P <0.5 ng/ml group was shown in **Figure 3B**. As serum P level was in the 1.00-1.24 ng/ml interval, CPRs showed a statistically noticeable improvement. However, when P levels were ≥2.00 ng/mL, CPRs showed a statistically significant decrease. Baseline characteristics and clinical outcomes between subgroups with different progesterone levels were compared in the normal weight population (**Supplemental Table 1**) and the overweight /obese population (**Supplemental Table 2**). In the normal weight population (**Supplemental Table 1**), basal FSH levels, basal E2 levels and basal P levels did not differ significantly between the two subgroups. Female BMI levels were lower in the P≥1ng/ml subgroup than in the P<1ng/ml subgroup. When serum P was elevated, the number of oocytes retrieved and the serum E2 levels were increased; while high-quality embryo rates, implantation rates, CPRs and live birth rates were significantly decreased. The results were similar for the overweight /obese population (**Supplemental Table 2**); however, high-quality embryo rates and live birth rates were not associated with serum P levels significantly.

To further explore the relationship between serum P on trigger day and CPRs, a multivariate logistic regression analysis was conducted utilizing regulations of Female age, Female BMI, AFC, infertility duration, basal E2, basal FSH, AMH, Gn dosage, days of stimulation, E2 on trigger day, P on trigger day, endometrial thickness on ET days, and the number of embryos transferred (**Table 3**). In the overweight/obese group, female age was negatively correlated with CPRs (OR: 0.935 [0.913-0.957], *p*<0.001), while the number of embryos transferred was positively related to CPRs (OR: 3.560 [2.763-4.588], *p*<0.001). The results for the normal weight group were similar (all *p*<0.05); however, CPRs were also negatively correlated with serum P on hCG day. In the normal weight group, an increase in serum P by one ng/mL was related to a 0.755 times lower probability of clinical pregnancy (95% CI: 0.677–0.841, *p* < 0.001).

**Table 3:** Association between clinical pregnancy and serum P level on the day of hCG by multivariable logistic regression analysis according to BMI.

Factors	18.5≤BMI<25		BMI≥25	
	Adjusted OR (95% CI)	*p* value	Adjusted OR (95% CI)	*p* value
Female age (y)	0.954 (0.939-0.969)	<0.001	0.935 (0.913-0.957)	<0.001
Number of embryos transferred (n)	2.727 (2.289-3.249)	<0.001	3.560 (2.763-4.588)	<0.001
P on trigger day (ng/ml)	0.755 (0.677-0.841)	<0.001	0.842 (0.702-1.009)	0.063

Female age, Female BMI, infertility duration, AFC, basal FSH, basal E2, AMH, Gn dosage, days of stimulation, E2 on trigger day, P on treigger day, number of embryos transferred, endometrial thickness on ET days were included in the multivariable regression model.

## Discussion

The study found that serum P levels on hCG trigger day are progressively lower as female BMI increases, and female BMI has an obvious moderating influence on the relationship between P level on triggering day and clinical pregnancy. In the normal weight group, a progesterone level of 1.00 ng/ml on triggering day was suggested to play the threshold role, which had an adverse effect on CPRs. In the overweight/obese group, low CPRs were not associated with PE until progesterone levels were greater than 2.00 ng/ml. Among all included cycles, that a boosted serum P level on triggering day correlated with a reduced CPR, as demonstrated by most previous studies (22, 27, 28). However, the multivariate logistic regression analysis stratified by BMI was only able to confirm this in normal weight cycles, indicating that the P level may serve as the predictor of adverse clinical pregnancy outcomes in the normal weight group except in cycles of overweight/obese women after taking the impact of other variables into consideration. These findings imply that CPRs should be considered in association with the PE and BMI.

In our study, we also demonstrated the detrimental influence of high female BMI on clinical outcomes in IVF/ICSI cycles, including more gonadotrophins required, longer stimulation days, lower 2PN oocyte rate, as well as lower high-quality embryo rate, which is consistent with previous studies (19, 20, 29, 30). Most importantly, baseline E2, FSH level, and E2 and P level on triggering day were significantly lower in the overweight/obese group compared to the normal weight group, consistent with findings from several prior studies (12, 31–34). For instance, previous research has reported that in cryopreserved blastocyst transfer cycles, serum P levels on the day of pregnancy testing were <15 ng/mL in 29% of women weighing >90.7 kg, versus 3% in those weighing <68 kg (32). In addition, in intrauterine insemination cycles for patients <35 years old, serum E2, LH, and P levels were negatively correlated with BMI on hCG day (33). However, it is worth noting that these studies had different inclusion criteria and included populations from different cycles. Grenman et al. (31) discovered that obese women had decreased levels of gonadotropins and sex steroids. Additionally, Santoro et al. (12) found that the concentrations of estradiol, P, LH and FSH metabolites were all lower in women with a BMI ≥25 kg/m² in a large epidemiological study including 848 women throughout the entire menstrual cycle. Furthermore, Jain et al. (13) collected the first-morning urine of 18 eumenorrheic women whose mean BMI was 48.6 kg/m^2^, seven of whom were in the early follicular stage. This group assessed hormone levels every 10 minutes to determine the pulsatile hormone-secreting rhythm, and the results showed that the mean P excretion and mean LH concentration were dramatically decreased in obese females. Leptin, which is produced by adipocytes, was found to be of promoting the function of the hypothalamus and maintain appropriate gonadotrophin secretion at appropriate physiological doses in normal weight adults. However, the high level of leptin in obese women can directly inhibit ovarian function, which was demonstrated to be of negative effect on LH and FSH output, ultimately influencing the level of P (35). All of the data demonstrated that obesity could alter the hypothalamic-pituitary-ovarian axis.

When discussing the exact endocrinologic mechanism of the impacts of PE on IVF/ICSI pregnancy outcomes, most studies have demonstrated the key role of endometrial receptivity. Epigenetic analysis of the endometrium also confirmed that PE on triggering day in IVF cycles altered epigenetic modification, like the expressions of H3K27me3 and H3k9me2, in three compartments of the endometrium during a pre-implantation period (9). Endometrial receptivity may be disrupted by altered epigenetic modification status. Through an electron microscope, George et al. (36) found that early elevated P levels can result in the premature formation of endometrial pinopodes and early shutdown of the transplantation window. This was consistent with our result of the reduced implantation rate and clinical pregnancy rate in women with high P on triggering day. Interestingly, as seen by a prospective randomized trial of 1205 parents with artificial endometrial preparation for ET, low P level on ET day was relevant to lower pregnancy outcomes (37), which implied that appropriate P levels were crucial for endometrial receptivity. And it may be an explanation of trends in CPRs when serum P levels <1.25ng/ml in the overweight/obese group.

Compared to non-obese women, obese women have significantly different endometrial transcriptomic profiles and are more likely to have altered endometrial gene expression, which may affect endometrial receptivity and result in lower implantation rates and more miscarriages (38). In our study, we found that a BMI in the range of ≥25 kg/m^2^ does not cause a poorer implantation rate. However, it was associated with a lower clinical pregnancy rate, a lower live birth rate, and an increased risk of miscarriage when compared to a normal BMI range, although the differences were not statistically significant. Our findings are consistent with some retrospective studies and reviews which suggest that obesity may detrimentally affect implantation and may increase the risk of miscarriage (17, 39). However, one study reported that overweight women transferring day 5-6 frozen-thawed blastocysts had a 16% higher transplant rate than normal-weight women (40). The divergent results across studies may be attributed to differences in protocols, Gn dosages, embryonic development stages, and embryo quality.

Interestingly, the normal weight and overweight/obese groups had different cutoffs for PE-induced impairment in our study. A higher P threshold concentration is related to a higher BMI. Since the normal weight group had a sufficient sample size (n=6368), the cutoff level (1.0 ng/ml) in the normal weight group may be a reasonable representation of the thresholds for the general population with cleavage embryo transfers (D3), similar to recent studies and a meta-analysis of over 60000 cycles. In contrast, the cutoff level (2.00 ng/ml) of the overweight/obese group is likely to be limited due to the smaller sample size (n =2628). Moreover, the outcome of the overweight/obese ET group tended to be related to multiple factors, especially oocyte and embryo quality, the number of embryos transferred, and female age, while endometrial receptivity may have a secondary effect. The analysis above may also explain why serum P on hCG day was negatively associated with CPRs in the overweight/obese group.

This study investigates the predictive value of serum P on clinical pregnancy in relation to female BMI, providing novel insights into the potential clinical utility of serum P for different BMI groups. Another strength of our study is that we utilized trend analysis and multivariate regression analysis to account for the nonlinear relationship between P levels and CPRs, reducing the possibility of bias. However, one of the limitations of our study is that the sample size of obese females was small, and there is significant difference in infertility duration between the two groups, which may explain some non-significant results that would be significant if more parents were included in each BMI population, especially the obese population. Additionally, the study only included fresh cycles with GnRH agonists and did not consider other COS protocols or frozen embryo transplantation. Finally, this work was a retrospective design with inevitable biases for patient selection.

## Conclusions

In summary, this study shows that women with higher BMI exhibited a lower P level on triggering day. Additionally, PE on hCG day is related to decreased CPRs in fresh IVF/ICSI cycles with cleavage embryo transfers regardless of women’s BMI level (normal weight VS overweight/obesity). PE has a progressive effect on CPR in the normal weight group starting at values of 1.00 ng/ml, but the effects become apparent in the overweight/obese group only starting from values ≥2.00 ng/ml. Our findings suggest that when PE is used as a predictor of clinical pregnancy outcomes, the threshold should be higher in the obese population compared to the normal-weight population. This highlights the importance of taking into account BMI when evaluating the effect of PE on triggering day on clinical pregnancy outcomes. The conclusion needs to be confirmed by prospective cohort studies with longer follow-ups and larger datasets, which may help to predict clinical pregnancy outcome of obese women.

## Data availability statement

The raw data supporting the conclusions of this article will be made available by the authors, without undue reservation.

## Ethics statement

The study has been reviewed and approved by the Ethics Committee of the First Affiliated Hospital of Zhengzhou University. Ethical approval number: 2022-87 KY-1099.

## Author contributions

QY and YS designed the research and guided writing. ZS and XL analyzed the data, made the table and drafted the manuscript. ZS, JX, YJ, WC collected the data. All authors contributed to the article and approved the submitted version.
