# Supplementary material for: Effect of BMI on the value of serum progesterone to predict clinical pregnancy outcome in IVF/ICSI cycles: a retrospective cohort study
Source: Front Endocrinol (Lausanne). 2023 Apr 19;14:1162302. doi: 10.3389/fendo.2023.1162302 (PMC10154690; doi:10.3389/fendo.2023.1162302)
Supplement: Supplementary file 1 [file Table_1.docx]

Supplementary Material

Effect of BMI on the value of serum progesterone to predict clinical pregnancy outcome in IVF/ICSI cycles: a retrospective cohort study

**Zhaoyang Shen^1,2,3†^, Xiaoyan Luo^1,2,3†^, Jianming Xu^1,2,3^, Yuqing Jiang^1,2,3^, Wenhui Chen^1,2,3^,** **Qingling Yang^1,2,3*^ and** **Yingpu Sun^1,2,3*^***

**Correspondence:** Yingpu Sun: [syp2008@vip.sina.com](mailto:syp2008@vip.sina.com) Qingling Yang: qingling531@163.com

**
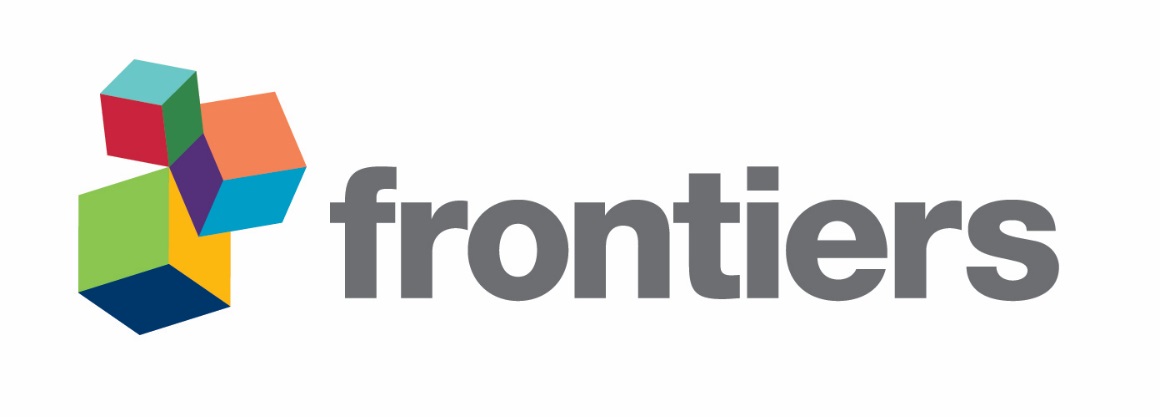
**

**Supplementary Table 1.** **Baseline characteristics and clinical outcomes** **between subgroups with different progesterone levels in normal weight parents**

|  | **P <1.00ng/ml** | **P ≥1.00ng/ml** | ***P* value** |
| --- | --- | --- | --- |
| **Variables** | **N=3530** | **N=2838** |  |
| **Female age (y)** | **30.3±4.1** | **31.2±4.2** | **<0.001** |
| **Female BMI (kg/m²)** | **21.8±1.7** | **21.7±1.7** | **＜0.001** |
| **Infertility duration (y)** | **3.7±2.7** | **3.7±2.7** | **0.660** |
| **AFC** | **14.7±6.4** | **12.9±5.6** | **＜0.001** |
| **Basal FSH (mIU/ml)** | **6.6±1.5** | **6.6±1.5** | **0.057** |
| **Basal E2(pg/ml)** | **42.0±25.8** | **43.0±24.6** | **0.143** |
| **Basal P (ng/ml)** | **0.5±1.1** | **0.5±1.3** | **0.753** |
| **AMH (ng/ml)** | **3.6±2.8** | **3.0±2.1** | **<0.001** |
| **Gn dosage (IU/L)** | **2388.7±1003.9** | **2669.8±888.7** | **＜0.001** |
| **Days of stimulation** | **13.3±2.2** | **13.6±1.8** | **＜0.001** |
| **E2 on hCG day (pg/ml)** | **2603.2±1342.4** | **3382.0±1631.3** | **＜0.001** |
| **P on hCG day (ng/ml)** | **0.6±0.2** | **1.5±0.4** | **＜0.001** |
| **Endometrial thickness on ET days (cm)** | **12.5±2.7** | **12.3±2.8** | **0.007** |
| **Oocytes retrieved** | **11.0±4.6** | **12.4±4.6** | **<0.001** |
| **2PN oocyte rate (%)** | **61.9% (23312/37660)** | **62.2% (20710/34299)** | **0.422** |
| **Cleavage rate of 2PN oocytes (%)** | **98.6% (22982/23312)** | **98.6% (20413/20710)** | **0.8** |
| **High-quality embryo rate (%)** | **64.7% (15073/23312)** | **62.5% (12947/20710)** | **＜0.001** |
| **Number of embryos transferred (n)** | **1.89±0.31** | **1.89±0.32** | **0.406** |
| **Implantation rate (%)** | **50.6% (3382/6679)** | **44.3% (2373/5351)** | **＜0.001** |
| **Clinical pregnancy rate (%)** | **69.5% (2455/3530)** | **62.3% (1767/2838)** | **<0.001** |
| **Live birth rate (%)** | **60.2% (2125/3530)** | **54.1% (1536/2838)** | **<0.001** |

**Supplementary Table 2.** **Baseline characteristics and clinical outcomes between subgroups with different progesterone levels in overweight and obese parents**

|  | **P <2.00ng/ml** | **P ≥2.00ng/ml** | ***P* value** |
| --- | --- | --- | --- |
| **Variables** | **N=2526** | **N=102** |  |
| **Female age (y)** | **30.9±4.3** | **31.5±4.3** | **0.184** |
| **Female BMI (kg/m²)** | **27.3±1.9** | **27.0±1.7** | **0.090** |
| **Infertility duration (y)** | **4.3±3.1** | **4.7±3.2** | **0.251** |
| **AFC** | **15.4±6.7** | **13.3±5.8** | **0.001** |
| **Basal FSH (mIU/ml)** | **6.3±1.5** | **6.3±1.3** | **0.913** |
| **Basal E2(pg/ml)** | **38.8±28.1** | **36.6±24.9** | **0.382** |
| **Basal P (ng/ml)** | **0.5±1.0** | **0.4±0.4** | **0.640** |
| **AMH (ng/ml)** | **3.5±2.7** | **2.8±1.6** | **<0.001** |
| **Gn dosage (IU/L)** | **2983.6±1011.6** | **3100.5±863.3** | **0.250** |
| **Days of stimulation** | **13.8±2.5** | **14.0±1.8** | **0.382** |
| **E2 on hCG day (pg/ml)** | **2380.1±1316.2** | **3039.7±1433.6** | **＜0.001** |
| **P on hCG day (ng/ml)** | **0.8±0.4** | **2.4±0.3** | **＜0.001** |
| **Endometrial thickness on ET days (cm)** | **12.5±2.8** | **13.1±2.8** | **0.031** |
| **Oocytes retrieved** | **11.5±4.8** | **14.3±4.8** | **<0.001** |
| **2PN oocyte rate (%)** | **60.0% (17101/28492)** | **55.6% (795/1429)** | **0.001** |
| **Cleavage rate of 2PN oocytes (%)** | **98.6% (16859/17101)** | **98.5% (783/795)** | **0.98** |
| **High-quality embryo rate (%)** | **62.2% (10642/17101)** | **60.9% (484/795)** | **0.443** |
| **Number of embryos transferred (n)** | **1.86±0.35** | **1.93±0.25** | **0.007** |
| **Implantation rate (%)** | **48.3% (2270/4697)** | **39.1% (77/197)** | **0.011** |
| **Clinical pregnancy rate (%)** | **65.7% (1660/2526)** | **61.0% (52/102)** | **0.002** |
| **Live birth rate (%)** | **55.6% (1405/2526)** | **49.0% (50/102)** | **0.189** |
